# Supplementary material for: WordCommentsAnalyzer: A windows software tool for qualitative research
Source: F1000Res. 2018 Sep 4;7:536. Originally published 2018 May 3. [Version 2] doi: 10.12688/f1000research.14819.2 (PMC6137411; doi:10.12688/f1000research.14819.2)
Supplement: Supplementary file 3 [file f1000research-7-17614-s0002.tgz › b2a79496-4ada-47c4-9682-a99ce867836d.zip!/Eight Iranian Health Journals/Archives of Rehabilitation_63.docx]

© Archives of Rehabilitation. This work is licensed under CC BY-NC 4.0 (https://creativecommons.org/licenses/by-nc/4.0/).

Volume & Issue: دوره 4 - شماره 3 و 4

Title: مقایسه اثر ایمونوگلوبولین وریدی با دُز بالا با ایمونوگلوبولین وریدی با دُز پایین در بیماران مبتلا به سندرم گیلن باره حاد

http://rehabilitationj.uswr.ac.ir/article-1-2-fa.html

هدف: سندرم گیلن باره حاد شایعترین علت پریفرال نوروپاتی یا واسطه ایمنی در اطفال است و با از‌بین‌رفتن پولیومیلیت در بیشتر کشورها، هم‌اکنون شایع‌ترین علت فلج شل حاد در کودکان است. بنابراین دست‌یابی به طریقه درمانی معتبر و مناسب از اهمیت بسزایی برخوردار است. در زمینه درمان‌های اختصاصی تاکنون تحقیقات گسترده‌ای روی کورتیکوستروئید، IVIG و پلاسمافورزیس انجام شده است. درمان با IVIG برای کودکان بی‌خطر و در گیلن باره حاد (GBS) ایمن‌تر و نسبت به روش‌های درمانی دیگر کمتر تروماتیک است. روش درمانی به دو شکل قابل اجرا است: 1- IVIG 1gr./kg/day به‌مدت 2 روز که اصطلاحاً High Dose گفته می‌شود؛ 2- IVIG 400mg./kg/day به‌مدت 5 روز که اصطلاحاً نوع Low Dose گفته می‌شود. در مطالعات خارجی نشان داده است که در کودکانی که High Dose IVIG دریافت کرده بودند طول مدت بیماری کوتا‌ه‌تر است. ازآنجاکه در ایران تا به حال بررسی مقایسه‌ای بین این دو روش صـورت نگرفتــه است، دو روش درمـانی را مـقایسه می‌کنیم و درصـورتی‌که IVIG با دز بالا اثرات مطلوب‌تر و پاسخ درمانی بهتری داشته باشد، به‌عنوان درمان انتخابی معرفی می‌نماییم. روش بررسی: بیمارانی که با شکایت ضعف اندام‌ها به‌صورت حاد به مرکز آموزشی درمانی مفید مراجعه نمودند و با توجه به معاینات بالینی، آزمایشات خون، مایع نخاع و انـجام EMG-NCV بیماری آنها گیلن باره حاد یـا Acute Demyelinating Peripheral Neuropathy تشخیص داده شد، وارد مطالعه گردیدند و به‌صورت تصادفی به دو گروه تجربی و شاهد تقسیم شدند. 25 بیمار گروه مطالعه توسط (روش High Dose) و 25 بیمار گروه شاهد توسط (روش Low Dose) بررسی شدند و سپس نتایج با هم مقایسه گردید. یافته‌ها: در این بررسی به این نتیجه مهم دست یافتیم که با به‌کارگیری روش درمانی ایمونوگلوبولین وریدی با دز بالا تعداد روزهای بستری بیماران کاهش می‌یابد و بیمار در مدت زمان کمتری بهبودی یابد. همچنین در این روش درمانی نسبت به روش درمانی ایمونوگلوبولین وریدی با دز پایین، بیمار علائم بهبودی را سریع‌تر نشان می‌دهد؛ بنابراین بیماران با عوارض دارویی کمتری روبه‌رو خواهند شد. نتیجه‌گیری: مدت زمان ضعف اندام تحتانی و فوقانی و مدت زمان بازگشت واکنش‌های اندام تحتانی و فوقانی با استفاده از این روش کاهش یافت و هیچ‌گونه ارتباطی بین عوارض مصرف داروی بیشتر با روش درمانی ایمونوگلوبولین وریدی با دز بالا مشاهده نشد.

Title: مقایسه توان‌بخشی به دو روش حرکت فعال زودهنگام و حرکت غیرفعال کنترل‌شده پس از ترمیم تاندون‌های فلکسور انگشتان دست در زون 2

http://rehabilitationj.uswr.ac.ir/article-1-3-fa.html

هدف: ترمیم تاندون‌های فلکسور در زون 2 دست، علی‌رغم پیشرفت‌های علمی در مورد آناتومی بیومکانیک، تغذیه، التیام تاندون‌ها و تکنیک‌های جراحی با مشکلاتی نظیر چسبندگی، کاهش لغزش تاندونی و نهایتاً محدودیت حرکتی انگشتان رو‌به‌روست. چسبندگی‌های محدودکننده که از لغزش تاندون جلوگیری می‌کنند علت رایج شکست این التیام‌هاست. نزدیک دو دهه است که روش توان‌بخشی حرکت اکتیو زودهنگام به‌همراه تکنیک جراحی مناسب توانسته تا حدی نتایج رضایت‌بخشی در ترمیم تاندونی این منطقه حاصل کند. هدف از مطالعه حاضر مقایسه دو روش EAM و CPM بعد از ترمیم‌های تاندونی در زون 2 است. روش بررسی: مطالعه مداخله‌ای حاضر روی 50 انگشت که توسط یک جراح و به روش (Strand Locked Cruciate 4) تحت ترمیم تاندون‌های فلکسوری در زون 2 قرار گرفته بودند و به مرکز تخصصی توان‌بخشی دست ایران (صبا) ارجاع شده بودند، انجام شد. نمونه‌گیری به‌صورت نمونه‌گیری قابل دسترس و تخصیص به دو گروه به‌صورت تصادفی انجام شد. دو گروه از نظر نداشتن ضایعات همراه و زمان ترمیم همسان بودند. دو روش توان‌بخشی «حرکت اکتیو زودهنگام» (EAM) و «حرکت پاسیو کنترل‌شده» (CPM) طبق پروتکل ذکرشده در متن انجام شد. یافته‌ها: بر اساس معیار Strickland در گروه اکتیو نتایج به صورت %44 عالی، %36 خوب، %20 متوسط و هیچ مورد ضعیف حاصل گردید. درصورتی‌که گروه پاسیو، %8 نتایج عالی، %32 خوب، %44 متوسط و %5/16 ضعیف بودند. براساس معیار Buck-Gramcko، در گروه اکتیو نتایج %12 عالی، %40 خوب، %32 متوسط و %16 ضعیف حاصل گردید. درصورتی‌که در گروه پاسیو %4 عالی، %12 خوب، %24 متوسط و %60 ضعیف بودند. میانگین TAM در گروه اکتیو 150/2 و در گروه پاسیو 116/41 بود که نشانگر بالاتربودن میزان لغزش تاندون‌ها در گروه اکتیو بوده است. نتیجه‌گیری: حرکت اکتیو زودهنگام با جلوگیری از ایجاد چسبندگی‌های محدودکننده ایجاد التیام تاندونی عمدتاً به روش (اینترنسیک)، تغذیه بهتر و ترمیم قوی‌تر روش مناسبی جهت توان‌بخشی بعد از ترمیم‌های تاندونی زون 2 است، مشروط بر آنکه تکنیک جراحی مناسبی جهت تقویت ترمیم تاندون‌ها به‌‌کار برده شود.

Title: بررسی اعتبار و تکرارپذیری اندازه‌گیری کیفوز پشتی ستون فقرات توسط خط‌کش انعطاف‌پذیر در افراد مبتلا به هیپرکیفوز وضعیتی

http://rehabilitationj.uswr.ac.ir/article-1-4-fa.html

هدف: افزایش کیفوز ستون فقرات بدلایل وضعیتی یکی از مشکلاتی است که برای ارزیابی و درمان آن نیاز به اندازه‌گیری دقیق مقدار زاویه کیفوز وجود دارد. خط‌کش انعطاف‌پذیر از ابزارهایی است که می تواند به‌طریق غیرتهاجمی مقدار کیفوز را اندازه بگیرد ولی تاکنون هیچ مطالعه‌ای، اعتبار و تکرارپذیری اندازه ‌گیری آن را در هیپرکیفوز وضعیتی بررسی نکرده است. هدف این مطالعه بررسی اعتبار و تکرارپذیری اندازه‌گیری کیفوز پشتی در افراد مبتلا به هیپرکیفوز وضعیتی است. روش بررسی: مقدار کیفوز پشتی در 25 فرد مبتلا به هیپرکیفوز وضعیتی و 15 فرد سالم 2 بار به‌وسیله خط‌کش انعطاف‌پذیر توسط یک آزمونگر اندازه‌گیری شد. همچنین مقدار کیفوز پشتی بر روی عکس رادیوگرافی دو بار توسط آزمونگر و یک بار توسط پزشک متخصص در افراد کیفوتیک اندازه‌گیری شد و تکرارپذیری اندازه‌گیری‌ها در دو گروه و همینطور اعتبار اندازه‌گیری خط‌کش انعطاف‌پذیر در مقایسه با عکس رادیولوژی در افراد بیمار بررسی گردید. یافته‌ها: تکرارپذیری Intratester اندازه‌گیری کیفوز توسط خط‌کش در هر دو گروه سالم و بیمار عالی گزارش گردید. همچنین اندازه‌‌گیری مقدار کیفوز بر کلیشه رادیوگرافی نیز تکرارپذیری Intertester و Intratester عالی داشت. بررسی اعتبار اندازه‌گیری کیفوز با خط‌کش برای اندازه‌گیری زاویه cobb روی قوس حاصل از خط‌کش ICC بسیار خوب داشت ولی برای اندکس کیفوز که روش قدیمی‌تری است، دارای مقادیر ضعیف بود. نتیجه‌گیری: خط‌کش انعطاف‌پذیر می‌تواند به‌عنوان وسیله‌ای معتبر و تکرارپذیر برای اندازه‌گیری غیرتهاجمی قوس کیفوز در افراد سالم و بیماران مبتلا به هیپرکیفوز وضعیتی پیشنهاد گردد.

Title: بررسی‌ اثر بکلوفن‌ در سم‌زدایی‌ بیماران‌ وابسته‌ به‌ تریاک‌

http://rehabilitationj.uswr.ac.ir/article-1-148-fa.html

هدف: نتایج بسیاری از مطالعات مؤید تأثیر احتمالی بکلوفن به‌عنوان آگونیست رسپتور گابا در سم‌زدایی از تریاک است. پژوهش حاضر مطالعه‌ای است که به‌منظور تعیین اثربخشی درمان کمکی با بکلوفن در سم‌زدایی از تریاک انجام شده است. روش بررسی: پژوهش حاضر مطالعه‌ای از نوع کارآزمایی بالینی با روش دوسوکور است. از بین کلیه بیماران مراجعه‌کننده با ملاک‌های وابستگی به تریاک براساس تسخه چهارم راهنمای تشخیصی و آماری اختلالات رواتی تعداد 52 بیمار ضمن مطابقت با ملاک‌های شمول موردنظر به‌صورت تصادفی و یک در میان در دو گروه درمانی جداگانه قرار گرفتند. علاوه‌بر رژیم ثابت درمانی در هر دو گروه به گروه اول بکلوفن به‌میزان 40 میلی‌گرم و به گروه دوم دارونما با ملاحظات دقیق پژوهشی و به‌صورت سرپایی و برای مدت دو هفته تجویز گردید. در هر دو گروه فرم کوتاه پرسشنامه ترک اعتیاد و پرسشنامه‌های خودساخته سایر نشانه‌های جسمی و نیز نشانه‌های روانی ترک اعتیاد در روزهای 0، 2، 4، 7، 14 پژوهش تکمیل گردید. یافته‌ها: در مقایسه دو گروه، بکلوفن اثربخشی نسبی بالاتری نسبت به دارونما در درمان علائم ترک وابستگی به تریاک نشان داد، ولی این تفاوت از نظر آماری معنادار نبود. نتیجه‌گیری: در مجموع به‌نظر می‌رسد اضافه‌کردن بکلوفن به رژیم درمانی سرپایی بیماران وابسته به تریاک شاید تا حد قابل قبولی در تخفیف نشانه‌های جسمی و تا حدی روانی ترک اعتیاد کمک نماید، گرچه تأکید بر این امر مستلزم کارآزمایی‌های تکمیلی بعدی است.

Title: بررسی اثر انتقال متقابل تمرینات یک‌طرفه عضله چهارسر ران به اندام مقابل

http://rehabilitationj.uswr.ac.ir/article-1-149-fa.html

هدف: وقتی یک اندام به هر دلیل بی‌حرکت می‌شود، ضعف و کاهندگی (آتروفی) عضلانی در اثر بی‌حرکتی و عدم استفاده از عضلات، مشکلات متداولی است که درمانگرها با آن مواجه هستند و روش‌های مختلفی برای پیشگیری و برطرف‌نمودن آن پیشنهاد شده است. از جمله جالب‌ترین آنها بهره‌گیری از پدیده انتقال متقابل است که به‌معنی تمرین‌دادن یک اندام و اثرگذاری بر اندام مثقابل است. هدف ما در این مطالعه، بررسی پدیده انتقال متقابل قدرت و تحمل ناشی از تمرینات عضله چهارسر یک اندام به اندام مقابل بوده است. روش بررسی: 46 نفر دانشجوی مذکر داوطلب در دو گروه آزمایش و کنترل در این مطالعه شرکت داشتند. نیمی از آنها (23 نفر) که گروه آزمایش بودند بعد از ارزیابی اولیه، تمرینات تجویزشده را با پای راست انجام دادند و پس از سه هفته تمرین مورد ارزیابی مجدد و نهایی قرار گرفتند. 23 نفر دیگر که در گروه کنترل قرار داشتند پس از ارزیابی، به‌مدت سه هفته یه زندگی عادی خود ادامه دادند و سپس مورد ارزیابی پایانی قرار گرفتند. یافته‌ها: نتایج به‌دست‌آمده پس از انجام آزمون روی اطلاعات و داده‌های به‌دست‌آمده حاکی از آن است که در گروه آزمایش متغیرهای قدرت و تحمل در هر دو پای تمرین‌دیده و تمرین‌ندیده دستخوش افزایش شده‌اند و میانگین تغییرات در هر دو پا یکسان است. درحالی‌که در گروه کنترل تغییرات معنی‌داری برای متغیرهای فوق ایجاد نشد. نتیجه‌گیری: با این نتایج وجود پدیده انتقال متقابل تأیید می‌شود و نظر به اینکه تغییرات در هر دو پای تمرین‌دیده و تمرین‌ندیده یکسان بوده است، می‌توان نتیجه گرفت در اوایل تمرین بروز تغییرات بیشتر به سازوکارهای عصبی مربوط است و سیستم اعصاب مرکزی به‌طور طبیعی برای حفظ تقارن این تغییرات را دو طرفه ایجاد می‌نماید.

Title: بررسی‌ تأثیر تمرین‌ ذهنی‌ بر تعادل‌ سالمندان‌

http://rehabilitationj.uswr.ac.ir/article-1-150-fa.html

هدف: تمرین‌ ذهنی‌ به‌‌معنای «مرور نمادین یک‌ فعالیت‌ فیزیکی‌ بدون‌ ایجاد حرکت‌ عضلانی‌ واضح‌» است. مطالعات‌ بسیاری‌ نشان‌ داده‌اند که‌ تمرین‌ ذهنی‌ مشابه‌ تمرین‌ فیزیکی‌ باعث‌ پیشرفت‌ مهارت‌ حرکتی‌ در افراد می‌شود؛ بنابراین‌ این‌ روش‌ درمانی‌ را می‌توان‌ به‌منظور توان‌‌بخشی‌ سالمندان‌ به‌کار برد. هدف‌ این‌ مطالعه‌ بررسی‌ تأثیر تمرین‌ ذهنی‌ بر یادگیری‌ مهارت‌ حرکتی‌ و تعادل‌ سالمندان‌ است‌. روش بررسی: این‌ پژوهش‌ روی‌ 30 نفر از سالمندان‌ سالم‌ بالای‌ 60 سال‌ در دو آسایشگاه‌ سالمندان‌ در شهر تهران‌ (آسایشگاه‌های‌ کهریزک‌ و فرزانگان‌) انجام‌ شد. افراد به‌‌صورت‌ تصادفی‌ به‌ دو گروه‌ آزمایش‌ و کنترل‌ تقسیم‌ شدند. تعادل‌ سالمندان‌ در چهار مرحله‌ مقدماتی‌، قبل‌ از درمان، پس‌ از درمان و پیگیری ارزیابی‌ گردید. سالمندان‌ در گروه‌ آزمایش‌ به‌‌مدت‌ سه‌ هفته‌ به‌ تمرین‌ ذهنی‌ یک‌ مهارت‌ حرکتی‌ و تعادلی‌ پرداختند و گروه‌ کنترل‌ در همان‌ دوره‌ هیچ‌ تمرین‌ خاصی‌ انجام‌ ندادند. یافته‌ها: نتایج پژوهش حاضر نشان می‌دهد که نمره آزمونهای عملکردی بالینی تعادلی پس از یک دوره تمرین ذهنی، تغییر معنی‌داری در افزایش نمره داشته است. نتیجه‌گیری: از نتایج پژوهش می‌توان چنین نتیجه گرفت که‌ تمرین‌ ذهنی‌ نه‌‌تنها روی‌ مهارت‌ اجراشده‌ به‌‌صورت‌ ذهنی‌ مؤثر بوده‌ است‌، بلکه‌ به‌‌صورت‌ معنی‌‌داری‌ باعث‌ پیشرفت‌ و بهبود تعادل‌ سالمندان‌ شده‌ است‌.

Title: مطالعه اثر زانوبند نئوپرنی و بانداژکشی روی درد و حس وضعیت مفصلی در بیماران مبتلا به استئوارتریت زانو

http://rehabilitationj.uswr.ac.ir/article-1-186-fa.html

هدف: هدف از این پژوهش تعیین تأثیر زانوبند نئوپرنی روی حس وضعیت مفصلی و درد بیماران استئوارتریت یک‌طرفه زانو و مقایسه میزان تأثیر آنها بود. در یک مطالعه شبه‌تجربی 30 نفر (11 مرد، 19 زن) از افراد مبتلا به استئوارتریت یک‌طرفه زانو با سنین 75-33 سال مورد آزمایش قرار گرفتند. درد بیماران براساس VAS قبل از آزمایش می‌بایست cm2 از cm10 باشد. روش بررسی: پای بیماران در حالت نشسته، به‌طور فعال در زاویه بین 60-30 درجه قرار گرفت (زاویه هدف) و بعد از 5 ثانیه مکث با چشمان بسته و دست‌به‌سینه این زاویه را چهار مرتبه بازسازی کردند. بازسازی اول جهت آشنایی بیمار بود. تفاضل میانگین سه دفعه بعد با زاویه هدف جهت مقایسه استفاده شد. برای تعیین میزان درد بیماران از معیار سنجش بصری (VAS) استفاده شد. (بدون درد=cm0 و درد شدید=cm10). یکی از مداخله‌ها بطور تصادفی بر روی پای بیماران بسته شد و مراحل فوق قبل و بعد از 20 دقیقه تکرار گردید . یک هفته بعد مداخله بعدی انجام شد. یافته‌ها: با استفاده از آزمون تی زوج‌شده، علامت‌های رتبه‌دار ویلکسون و ضریب پیرسون داده‌ها مورد تجزیه و تحلیل قرار گرفت و نشان داد که میانگین خطای زاویه‌ای قبل و بعد از بستن زانو بند نئوپرنی تفاوت معنی‌داری دارد (P=0/037) و زانوبند حس وضعیت مفصلی را افزایش داده است، ولی بانداژ اثری روی حس وضعیت مفصلی نداشته (0/631=P). در مورد درد بیماران، هم بانداژ و هم زانوبند درد را به‌طور معنی‌دار کاهش دادند (0/000=P) که این اثر در مورد زانوبند بیشتر بوده است (0/001=P). نتیجه‌گیری: در این مطالعه میزان اثر زانوبند نئوپرنی بر حس وضعیت و درد بیماران مبتلا بیشتر از بانداژکشی بوده است. جهت تعیین تأثیر بانداژ پیشنهاد می‌شود که انواع دیگر بانداژ مورد بررسی قرار گیرند.

Title: تشخیص افتراقی لکنت اولیه و ناروانی طبیعی در کودکان

http://rehabilitationj.uswr.ac.ir/article-1-187-fa.html

هدف: تشخیص و مداخله زودهنگام لکنت اولیه، کلید پیشگیری از لکنت رشدی مزمن است. یکی از موارد مهم تشخیص افتراقی لکنت اولیه، ناروانی طبیعی کودکان زیر 5 سال است. هدف از تحقیق حاضر تعیین صحت و سقم تشخیص‌های داده‌شده در مورد کودکان مراجعه‌کننده به کلینیک گفتاردرمانی به‌عنوان ناروانی طبیعی و معرفی روش‌های دقیق‌تر تشخیص افتراقی است. روش بررسی: روش تحقیق حاضر از نوع پژوهشی است که طی آن با استـفاده از روش نمونه‌گیری آسان تعداد 10 کودک لکنتی مراجعه‌کننده به بخش گفتاردرمانی کلینیک توان‌بخشی صبا در سال‌های 1382 و 1383 مورد مطالعه قرار گرفتند. ابزار تحقیق و روش جمع‌آوری داده‌ها شامل پرسشنامه، ضبط نمونه گفتار خودبه‌خودی و به‌دست‌آوردن زمان VOT با استفاده از دستگاه لارنگوگراف بود. لازم به‌ذکر است که با توجه به نبود داده‌های هنجار در مطالعه VOT کودکان لکنتی، داده‌های VOT کودکان عادی همتاشده با آنان نیز مورد بررسی و مقایسه قرار گرفت. یافته‌ها: براساس یافته‌های این تحقیق که فقط یک مورد از کودکان براساس شاخص کمّی محاسبه‌شده ناروان طبیعی محسوب شد و سایر کودکان همگی مبتلا به لکنت اولیه بودند. همچنین داده‌های VOT کودکان لکنتی با کودکان عادی همتاشده مورد مقایسه قرار گرفت و تحلیل آماری نشان داد که VOT کودکان لکنتی به‌طور معنی‌دار (0/05>P) از کودکان عادی طولانی‌تر است. نتیجه‌گیری: یافته‌های تحقیق حاضر شاخص کمّی و کلینیکی مناسبی را در اختیار آسیب‌شناسان گفتار و زبان قرار می‌دهد تا به کمک آن بتوانند به‌طور معتبر بین لکنت اولیه و ناروانی طبیعی تشخیص افتراقی دهند. همچنین می‌توان از یافته‌های VOT کودکان در تکمیل تشخیص افتراقی بهره گرفت.

Title: بررسی اختلالات شنوایی در بیماران مبتلا به اسکلروز متعدد

http://rehabilitationj.uswr.ac.ir/article-1-188-fa.html

هدف: ام.اس یک بیماری شایع و مزمن نورولوژیک است که به‌عنوان یکی از علل کم‌شنوایی ناگهانی مطرح است. در این پژوهش اختلالات شنوایی افراد مبتلا به ام.اس مورد بررسی قرار گرفته است. روش بررسی: در این مطالعه توصیفی-تحلیلی مقطعی 107 بیمار میتلا به ام.اس در محدوده سنی 45-20 سال که هیچ‌گونه سابقه ضربه به گوش و مشکلات گوش میانی نداشتند، به‌صورت تصادفی انتخاب شدند و ارزیابی کامل شنوایی شامل ادیومتری صوت خالص، آزمون‌های گفتاری، ایمیتانس ادیومتری و پتانسیل‌های برانگیخته شنوایی ساقه مغزی (BAEP) روی آنها انجام شد. یافته‌ها: در آزمایش ادیومتری صوت خالص 21 نفر از مبتلایان به ام.اس (%19/62) دچار کاهش شنوایی بودند که بیشترین موارد از نوع کم‌شنوایی حسی عصبی در فرکانس‌های بالا بود. امتیازات بازشناسی گفتار و نتایج رفلکسومتری به‌ترتیب در 18 و 31 مورد غیرطبیعی بود. 59 بیمار از افراد مورد مطالعه (%55/14) دارای نتایج غیرطبیعی در آزمایش BAEP بودند که بیشترین اختلال به‌ترتیب افزایش زمان نهفتگی مطلق موج V، کاهش نسبت دامنه امواج &Iota/V و تکرارپذیری ضعیف امواج بود. با افزایش سرعت تحریک، پاسخ‌های غیرطبیعی به 83 مورد (%77/57) رسید. نتایج آزمون‌های آماری نشان داد بین سرعت تحریک و زمان نهفتگی موج V در آزمایش BAEP تفاوت معنی‌داری وجود دارد. نتیجه‌گیری: با توجه به یافته‌های این پژوهش به‌نظر می‌رسد ارزیابی شنوایی برای پیگیری اختلالات شنوایی و توان‌بخشی به‌موقع افراد مبتلا به ام.اس بسیار حائز اهمیت است. همچنین می‌توان از مجموعه آزمون‌های شنوایی به‌ویژه آزمایش BAEP با استفاده از سرعت تحریک بالا به‌عنوان ابزار کمکی برای تشخیص بیماری ام.اس بهره گرفت.

Title: تأثیرات خطاهای ناشی از جابه‌جایی نشانگرهای تحلیل حرکتی روی نقاط استخوانی در هنگام اندازه‌گیری وضعیت و راستای سه‌بُعدی استخوان کتف

http://rehabilitationj.uswr.ac.ir/article-1-189-fa.html

هدف: ضعف و خستگی عضلات اسکاپولوتوراسیک می‌تواند منتهی به غیرطبیعی‌شدن وضعیت کتف و به‌هم‌خوردن ریتم اسکاپولوهومرال و اختلال در عملکرد شانه شود. به‌دلیل آنکه استخوان کتف به‌صورت سه‌بُعدی حرکت می‌کند، تکنیک‌های دوبُعدی نمی‌تواند به‌طور کامل نمایانگر حرکت آن باشد. یکی از روش‌های نصب نشانگر در ارزیابی سه‌بُعدی حرکت، قراردادن آن روی پوست نقاط استخوانی است، درحالی‌که در هنگام حرکت اندام، جابه‌جایی نشانگر پوستی از روی نشانه استخوانی زیرش می‌تواند منشأ خطا در اندازه‌گیری پارامترهای کینماتیکی باشد. هدف از این تحقیق بررسی خطای ناشی از جابه‌جایی نشانگرهای دستگاه تحلیل حرکتی در اندازه‌گیری متغیرهای کینماتیکی کتف و همچنین تعیین تکرارپذیری این روش است. روش بررسی: به روش نمونه‌گیری غیراحتمالی ساده یک نمونه 10 نفری از افراد سالم با دستگاه Kinemetrix طی سه جلسه متوالی مورد آزمایش قرار گرفتند. پس از نصب نشانگرها روی زاویه تحتانی،‌ ریشه خار، اکرومیون و زوائد خاری مهره‌های هفتم گردنی و پشتی و زائده اوله ‌کرانون در وضعیتی که دست‌ها در کنار بدن آویزان است، اندام در هر وضعیت آزمون در زوایای ْ45 ، ْ90،  ْ120 و دامنه کامل قرار داده شد، نقاط استخوانی از طریق لمس پیدا و نشانگر مجدداً روی آن چسبانیده و تصاویر سه‌بُعدی نشانگرها طی یک ثانیه ثبت گردید. این پروسه در جلسه دوم نیز عیناً تکرار ‌شد. در جلسه سوم پس از نصب اولیه نشانگرها تا انتهای دامنه هیچ‌گونه جابه‌جایی در محل نشانگر صورت نگرفت. یافته‌ها: مقادیر میانگین ضرایب همبستگی متغیرهای مورد مطالعه در طی جلسات اول و دوم در دامنه 0/84-0/92 و در روش عدم تغییر نشانگر در دامنه 0/77-0/54 قرار می‌گیرد. نتیجه‌گیری:در اندازه‌گیری وضعیت و راستای سه‌بعدی استخوان کتف به‌علت جابه‌جاشدن نشانگرهای پوستی نصب‌شده روی نقاط استخوانی خطای قابل ملاحظه‌ای در اندازه زوایای چرخش به‌سمت بالا و تیلت خلفی کتف حاصل گردید.

Title: الکترومیوگرافی بدون درد در کودکان

http://rehabilitationj.uswr.ac.ir/article-1-190-fa.html

امروزه به کمک تکنیک‌های پیشرفته ژنتیک مولکولی، اولتراسون و MRI بسیاری از بیماری‌های اعصاب و عضلات در کودکان تشخیص داده می‌شوند. این روش‌ها در مقایسه با الکترومیوگرافی هم حساس‌ترند و هم درد کمتری دارند؛ بنابراین تکنیک EMG هنوز با توجه به سهولت دسترسی و قدرت تمایز بالا در بیماری‌های نخاع، اعصاب محیطی و عضلات در اطفال جایگاه مناسبی دارد. به‌هرحال این روش تشخیصی در کودکان به‌دلیل تحریک اعصاب محیطی با جریان‌های الکتریکی از یک‌سو و هم به‌دلیل استفاده از الکترود سوزنی در مطالعه عضلات درد ایجاد می‌کند. بر همین اساس تمام متخصصان این رشته به‌دنبال روش‌هایی هستند که با کم‌کردن درد به‌وجودآمده از EMG کارایی آن را افزایش دهند. در مقاله حاضر سعی شده است تا بعد از توضیح مختصر در مورد مراحل انجام الکترومیوگرافی در کودکان، به تکنیک‌هایی اشاره شود که با استفاده از آنها در هر مرحله می‌توان درد حاصله از انجام EMG را به حداقل رساند.

Title: اولین مورد گزارش‌شده از جهش در COL11A2 با ناشنوایی ارثی اتوزومال مغلوب غیرسندرمی در یک خانواده ایرانی

http://rehabilitationj.uswr.ac.ir/article-1-517-fa.html

کاهش شنوایی، در 1 نفر از 2000 کودک تازه متولدشده دیده می‌شود که بیش از 50 درصد این موارد را عوامل ژنتیکی دربرمی‌گیرد. کاهش شنوایی غیرسندرمی بیش از 70 درصد موارد ناشنوایی ارثی را شامل می‌شود که 85 درصد آن به شکل اتوزومال مغلوب منتقل می‌شود و وجود بیش از یک‌صد جایگاه ژنی (Locus) برای آن تخمین زده می‌شود. هدف در این مطالعه تعیین جهش ژنی در یک خانواده ناشنوای غیرسندرمی که دارای دو فرزند مبتلا به ناشنوایی هستند، بوده است. این بررسی قسمتی از طرح شناسایی ژن‌های شایع ناشنوایی ارثی غیرسندرمی اتوزومال مغلوب است. در این تحقیق پیوستگی بین ژن COL11A2 در لوکوس DFNA13 -که ایجادکننده ناشنوایی با وراثت اتوزومی غالب است- و بیماری در یک خانواده ایرانی مبتلا به ناشنوایی غیرسندرمی با وراثت اتوزومی مغلوب مورد بررسی قرار گرفت و برای اولین بار جهش Pro 621Thr در این خانواده ایرانی مبتلا به ناشنوایی غیرسندرمی با توارث نهفته شناسایی شد. پسربچه 4 ساله‌ای با ناشنوایی مادرزادی بدون وجود سایر علائم بالینی به‌منظور انجام آزمایش‌های ملکولی به مرکز تحقیقات ژنتتیک ارجاع داده شد. این پسربجه خواهر 16 ساله‌ای نیز ناشنوایی داشت و ازدواج پدرومادر ایشان از نوع ازدواج فامیلی درجه سوم بود. در ابتدا بیماران برای جهش‌های ژن GJB2 و GJB6 بررسی شدند و سپس به‌وسیله تحلیل پیوستگی برای یافتن محل ژن درگیر مورد مطالعه قرار گرفتند. بر اساس معاینات بالینی به‌عمل‌آمده، هر دو مورد ناشنوایی ارثی غیرسندرمی دارند که براساس شرح حال و رسم شجره،‌ نحوه توارث آن اتوزومی مغلوب است و جهش Pro 621Thr در ژن COL11A2 در بیماران تشخیص داده شد. خانواده فوق یکی از خانواده‌هایی است که برای شناسایی جهش‌های شایع ناشنوایی غیرسندرمی اتوزومی مغلوب مورد بررسی قرار گرفت. با تحلیل پیوستگی در خانواده مذکور جایگاه کروموزومی این خانواده مورد بررسی قرار گرفت که ناحیه 6p21-3 برای این خانواده مشخص شد. با انجام آزمون‌های ملکولی برای مورد و نیز خواهر وی در مرکز تحقیقات ژنتیک، دو جهش شایع GJB2 و GJB6 منفی بوده است. این جایگاه محل قرارگیری ژن COL11A2 است که جهش‌های متعددی در این ژن گزارش شده است. جهش در این ژن همچنین می‌تواند منجر به ایجاد سندرم استیکلر نوع 3 با توارث اتوزومال غالب و نیز ناشنوایی ارثی غالب غیرسندرمی گردد. جهش شناسایی‌شده جابه‌جایی Pro 621Thr است که اولین مورد گزارش‌شده از جهش در این ژن به‌صورت اتوزومی مغلوب و غیرسندرمی است.
